# Genotypic and Environmental Variations in Grain Cadmium and Arsenic Concentrations Among a Panel of High Yielding Rice Cultivars

**DOI:** 10.1186/s12284-017-0149-2

**Published:** 2017-03-28

**Authors:** Guilan Duan, Guosheng Shao, Zhong Tang, Hongping Chen, Boxun Wang, Zhu Tang, Yuping Yang, Yuechuan Liu, Fang-Jie Zhao

**Affiliations:** 1 State Key Laboratory of Urban and Regional Ecology, Research Centre for Eco-Environmental Sciences, Chinese Academy of Sciences, Beijing, 100085 China; 2 Chinese National Rice Research Institute, Hangzhou, 310006 China; 3 State Key Laboratory of Crop Genetics and Germplasm Enhancement, College of Resources and Environmental Sciences, Nanjing Agricultural University, Nanjing, 210095 China; 4 Youxian Agricultural Bureau of Hunan Province, Hunan, 412300 China

**Keywords:** Arsenic, Cadmium, Food Safety, Genotype, Rice

## Abstract

**Background:**

Rice is a major dietary source of cadmium (Cd) and arsenic (As) for populations consuming rice as the staple food. Excessive Cd and As accumulation in rice grain is of great concern worldwide, especially in South China where soil contamination with heavy metals and metalloids is widespread. It is important to reduce Cd and As accumulation in rice grain through selection and breeding of cultivars accumulating low levels of Cd or As.

**Results:**

To assess the genetic and environmental variations in the concentrations of Cd and As in rice grains, 471 locally adapted high-yielding rice cultivars were grown at three moderately contaminated sites in South China for two years. Cadmium and As concentrations in brown rice varied by 10 – 32 and 2.5 – 4 fold, respectively. Genotype (G), environment (E) and G x E interactions were highly significant factors explaining the variations. Brown rice Cd concentration was found to correlate positively with the heading date among different cultivars, whereas As concentration and heading date correlated negatively. There was a significant and negative correlation between grain Cd and As concentrations.

**Conclusions:**

Eight and 6 rice cultivars were identified as stable low accumulators of Cd and As, respectively, based on the multiple site and season trials. These cultivars are likely to be compliant with the grain Cd or As limits of the Chinese Food Safety Standards when grown in moderately contaminated paddy soils in South China.

**Electronic supplementary material:**

The online version of this article (doi:10.1186/s12284-017-0149-2) contains supplementary material, which is available to authorized users.

## Background

Cadmium (Cd) and arsenic (As) are both classified as a Group-1 carcinogen by the International Agency for Research on Cancer (IARC, 1993) and the National Toxicology Program (NTP 2000). Arsenic and Cd rank first and seventh, respectively, on the Agency for Toxic Substances and Disease Registry Priority List of Hazardous Substances (www.atsdr.cdc.gov/SPL/index.html). Long-term exposure to high levels of Cd may lead to a variety of health problems, including Itai-Itai disease (Murata et al., 1970; Bhattacharyya et al., 1992; Satarug and Moore, 2004), whilst long-term exposure to high levels of As may hurt the nervous system, damage blood vessels or lead to cancer of humans (Chen et al., 1986; Smith et al., 2000).

Cadmium and As are ubiquitous in the environment due to natural pedogenic processes, such as weathering of minerals, and to anthropogenic activities, such as mining, waste disposal, applications of fertilizers and agrochemicals (Nordstrom, 2002; Liu et al., 2005). A recent nationwide soil survey in China showed that 7 and 2.7% of the soil samples were contaminated with Cd and As, respectively (MEP, 2014). There is also evidence that average soil Cd concentrations have increased considerably over the last three decades, by over 50% in the coastal and southwest regions (Zhao et al., 2015).

Compared with other heavy metals, Cd has a relatively high bioavailability in soil and can be readily taken up by plants (Bolan et al. 2003; Clemens & Ma, 2016). Substantial proportions of rice grain produced in some areas in southern China were found to exceed the Chinese Food Hygiene Standards for Cd (0.2 mg kg^-1^) (Zhen et al., 2008; Zhu et al., 2008; GB2762, 2012). For example, Du et al. (2013) showed that 60% of the rice samples collected from a county in northern Hunan province exceed the 0.2 mg Cd kg^−1^ limit, with 11% of the samples containing >1.0 mg Cd kg^−1^. Williams et al. (2009) reported that 65% of rice grain samples from paddy fields impacted by mining activities in Hunan exceeded the Cd limit. Zhu et al. (2016) showed that 76% of rice grain samples from central-eastern Hunan province exceeded the Cd limit, with the maximum Cd concentration up to 4.8 mg kg^-1^. A number of factors likely contribute to the elevated Cd levels in rice grain in southern China, including contamination of soil and irrigation water, soil acidification and cultivation of rice cultivars with high Cd accumulation (Zhao et al. 2015; Zhu et al. 2016). Paddy rice is also efficient at accumulating As (Williams et al. 2007). This is because the anaerobic conditions in flooded paddy soil are conducive to the mobilization of arsenite (Xu et al. 2008; Arao et al., 2009), which is subsequently taken up by the highly expressed silicon uptake pathway in rice roots (Ma et al. 2008; Zhao et al. 2010). Arsenic accumulation in rice grain is further elevated by contamination of paddy soil or irrigation water (Zhu et al. 2008; Dittmar et al. 2010). For example, Zhu et al. (2008) and Williams et al. (2009) showed that 50–65% of the rice grain samples from mining-impacted paddy fields in Hunan province exceeded the Chinese Food Hygiene Standards for As of 0.15 mg kg^-1^ inorganic As (GB 2715-2005, which has been superseded by GB2762 (2012) with the limit of inorganic As raised to 0.2 mg kg^-1^).

Rice consumption constitutes a major source of dietary intake of inorganic As and Cd for populations whose staple food is rice (Tsukahara et al., 2003; Mondal & Polya, 2008; Signes-Pastor et al., 2016). Li et al. (2011) showed that rice contributes up to 50 and 60% of the total dietary inorganic As for the Bangladeshi and Chinese populations, respectively. For Cd, rice and its products also contribute up to 50% of the ingested Cd for most Asian populations (Tsukahara et al., 2003). Therefore, reducing Cd and As accumulation in rice is important for food safety and public health.

Depending on the soil conditions and the characteristics of the contaminant, a number of mitigation measures may be employed to reduce Cd and As accumulation in rice grains (Zhao et al. 2015; Chaney et al., 2016). These include liming of acidic soils, paddy water management, selection and breeding of rice cultivars with low accumulation of the contaminants, and phytoremediation. Liming is effective in decreasing Cd bioavailability in acidic soils and Cd uptake by rice (Bolan et al., 2003; Zhu et al. 2016), but not for As. Paddy water management can produce opposite effects on Cd and As accumulation. Maintaining flooded paddy conditions decreases Cd accumulation, but increases As accumulation in rice grains (Li et al. 2009; Arao et al. 2009; Hu et al. 2013). Phytoremdiation has been tested in small scale field trials (Murakami et al., 2009; Mandal et al., 2012; Deng et al., 2015), but its applicability to cleaning up large areas of contaminated paddy soils remains uncertain. Selection of cultivars accumulating low levels of Cd or As represents a feasible and practical option because there exist large genetic variations among rice cultivars in the accumulation of these metals and metalloids in grain (Norton et al., 2012a; Duan et al., 2012; Kuramata et al., 2013; Pinson et al. 2015). For example, Norton et al. (2012a) conducted six field trials in Bangladesh, China and USA over 2 years and found there was a 3–34 fold variation in grain As concentration among *c.* 300 rice accessions (Norton et al., 2012a). Pinson et al. (2015) reported 40.7 and 12.1-fold variations for grain Cd and As concentrations, respectively, among 1763 rice accessions of diverse geographic and genetic origin when grown under flooded paddy conditions. Pinson et al. (2015) also found that Cd and As variation had a significant genetic basis, with broad sense heritability varying from 0.24 to 0.63 for Cd and from 0.57 to 0.64 for As, respectively. These results indicate substantial and heritable genetic variability among rice varieties or germplasm that can be employed to reduce Cd and As accumulation in rice grain.

However, most of the previous screening studies used rice germplasm resources, and the accessions they found to have low levels of As or Cd accumulation may not be suitable to the growth conditions in South China. Therefore, these low accumulating accessions cannot be immediately usable in South China, where the problem of heavy metal and metalloid contamination is serious. In the present study, variations in grain Cd and As accumulation among 471 high yielding rice cultivars that are widely grown in southern China were investigated in two years at multiple sites with moderate levels of Cd contamination in Southern China. A number of cultivars with low Cd or As accumulation across multiple environments were selected. The relationships between grain Cd and As concentrations and heading date were examined.

## Results

Field experiments were conducted at three sites. Soils at all sites are acidic (Table 1). Two of the sites (Youxian and Fuyang) contain moderate levels of Cd (0.4 – 0.5 mg kg^-1^) (Table 1), both exceeding the Cd limit of the Chinese soil environmental quality standard (0.3 mg kg^-1^ for soils with pH < 7.5; GB 15618, 1995). The other site (Xiangtan) contains a higher level of Cd (1.4 mg kg^-1^) than the other sites. The total soil As concentrations were higher than the background level (<10 mg kg^-1^), but still below the As limit of the Chinese soil environmental quality standard (30 mg kg^-1^ for paddy soils) (GB 15618, 1995) (Table 1). In 2014, 471 rice cultivars widely grown in Southern China were grown at Youxian and Fuyang. Based on the results of the trials in 2014, 63 rice cultivars were selected for further trials at three sites in 2015.

**Table 1:** pH and heavy metal concentrations in soils at the experimental sites

Site	pH	As (mg kg^-1^)	Cd (mg kg^-1^)	Cu (mg kg^-1^)	Zn (mg kg^-1^)	Pb (mg kg^-1^)
Youxian (2014)	4.88	22.5	0.55	28.6	137.4	42.3
Youxian (2015)	5.19	17.4	0.46	29.9	100.7	35.4
Fuyang	5.64	12.2	0.39	28.3	120.6	36.4
Xiantan	4.87	19.4	1.40	27.4	125.3	39.9
Standards*		30	0.3	50	200	250

*National Environmental Protection Bureau Environmental quality standard for soils GB 15618, 1995

### Cadmium Concentration in Rice Grain

**Fig. 1:**
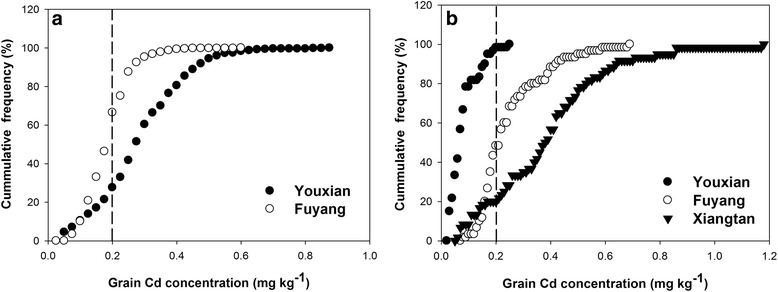
Cumulative frequency of Cd concentration in brown rice harvested at two sites in 2014 (**a**) and three sites 2015 (**b**). The vertical dash line indicates Chinese Food Hygiene Standards for rice Cd concentration (0.2 mg kg^-1^)

Out of the 471 cultivars (Additional file 1: Table S1) planted in 2014, 466 and 462 cultivars grew to maturity and were harvested successfully at the experimental sites at Youxian, Hunan Province and Fuyang, Zhejiang Province, respectively. Cadmium concentration in rice grain (i.e. unpolished brown rice) harvested from Youxian and Fuyang ranged from 0.03 to 0.86 mg kg^-1^ and from 0.06 to 0.58 mg kg^-1^, respectively (Fig. 1a), representing about 32 and 10 fold variation among the harvested cultivars. At the Youxian and Fuyang sites, 83 and 34%, respectively, of the harvested cultivars exceeded the Chinese Food Hygiene Standards (GB2762 2012, 0.2 mg kg^-1^ Cd in the grain).

Based on the results of 2014, 52 cultivars were further tested in 2015, including 44 low Cd, 3 high Cd and 5 low As rice cultivars. Additionally, 11 cultivars with late heading date were also included. Of the 63 cultivars planted in 2015, 62, 63 and 61 cultivars were successfully harvested from the Youxian, Fuyang and Xiangtan (Hunan Province) sites, respectively. Cadmium concentration in rice grain ranged from 0.02 to 0.24 mg kg^-1^, from 0.08 to 0.68 mg kg^-1^, and from 0.14 to 1.17 mg kg^-1^ at Youxian, Fuyang and Xiangtan, respectively (Fig. 1b). The variations among different cultivars were 10, 9 and 8 fold, respectively, with 2, 52 and 80% of the cultivars harvested exceeding the grain Cd limit. The much lower percentage of Cd exceedance in 2015 at the Youxian site, compared with the 2014 results, could be explained by the selection of 44 low Cd cultivars, a slightly lower soil Cd concentration and a higher pH in the field used in 2015 (Table 1), and a wetter season in 2015 than 2014 which could have resulted in a lower Cd bioavailability.

For each year, two-way ANOVA analysis on the pooled data from different sites indicated that grain Cd concentration was significantly affected by genotype (G), environment (E), and G × E interactions (Table 2). Genotype, E and G × E accounted for 48, 16, and 26%, respectively, of the total variation in 2014. The corresponding percentages in 2015 were 30, 41 and 28%, respectively. The higher contribution from G in 2014 than in 2015 could be explained by the much larger number of cultivars grown in the first year. In contrast, the 2015 trials included one additional site with a relatively higher level of soil Cd, thus resulting in a higher percentage of contribution from E to the total variation. In both years, the contribution of G × E interactions to the total variation was similar.

**Table 2:** Two-way ANOVA analysis of grain Cd and As concentrations in two sites in 2014 and three sites in 2015

	Variation	SS	df	MS	F^a^	F crit	*P*-value	SSE/SST (%)^a^
Cd								
2014	Genotype (G)	20586173	455	45244	20.00	1.13	<0.0001	48.46
	Environment (E)	6920449	1	6920449	3059.48	3.85	<0.0001	16.29
	G × E	10850810	455	23848	10.54	1.13	<0.0001	25.54
	Error	4125834	1824	2262				
2015	Genotype (G)	6047463	59	102499	123.55	1.36	<0.0001	29.65
	Environment (E)	8285986	2	4142993	4994.02	3.02	<0.0001	40.62
	G × E	5767217	118	48875	58.91	1.27	<0.0001	28.27
	Error	298653	360	830				
As								
2014	Genotype (G)	3579026	455	7866	6.33	1.13	<0.0001	38.24
	Environment (E)	1169753	1	1169753	941.51	3.85	<0.0001	12.50
	G × E	2344688	455	5153	4.15	1.13	<0.0001	25.05
	Error	2266179	1824	1242				
2015	Genotype (G)	1025771.997	59	17386	42.94	1.36	<0.0001	31.05
	Environment (E)	1096363.439	2	548182	1353.85	3.02	<0.0001	33.19
	G × E	1035829.127	118	8778	21.68	1.27	<0.0001	31.35
	Error	145765.79	360	405				

^a^Sum of squares (SS) of each effect by total SS

### Stability of Grain Cd Concentration Across Sites and Seasons

**Fig. 2:**
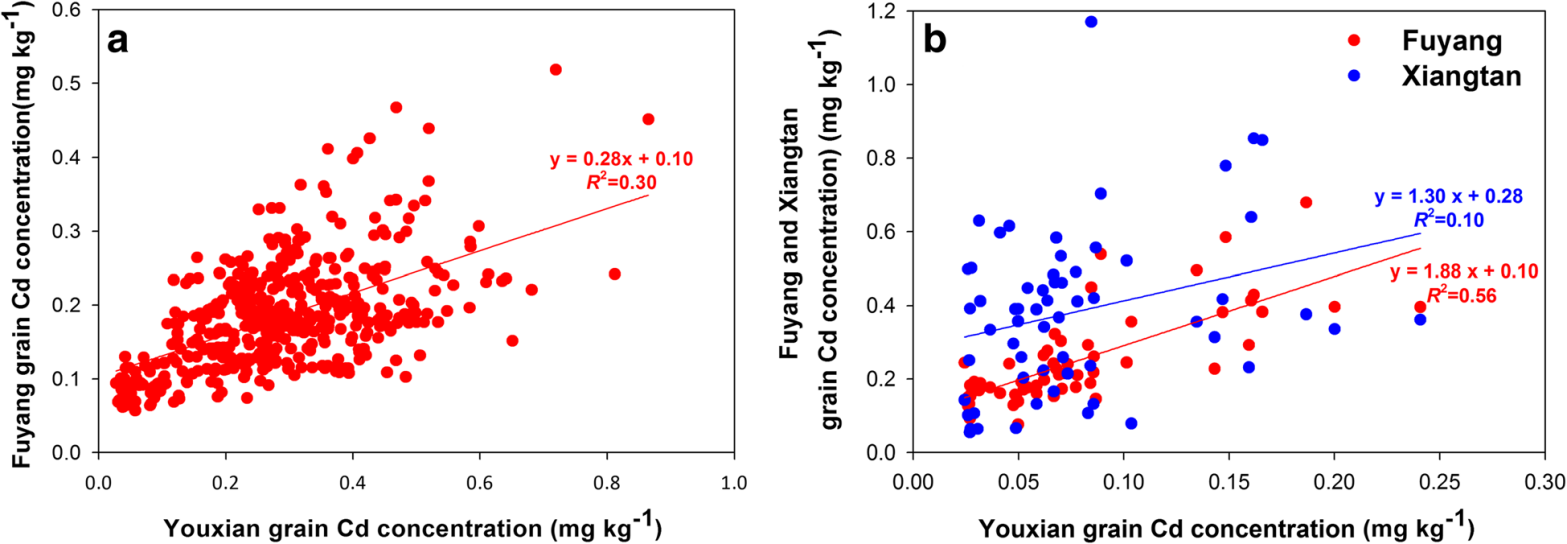
Correlation between grain Cd concentrations of the common cultivars grown at different sites in 2014 (**a**) and 2015 (**b**)

Correlation analysis was used to assess the stability of grain Cd concentration of rice cultivars across sites and years. Among the 456 common cultivars between Youxian and Fuyang in 2014, Cd concentration in brown rice correlated significantly (*r* = 0.55, *P* < 0.0001) (Fig. 2a, Additional file 1: Table S2). In 2015, there were 60 common cultivars harvested from the three sites. Significant correlations were found between each two of the three sites (Fig. 2b, Additional file 1: Table S2). The correlation was stronger between Youxian and Fuyang (*r* = 0.75, *P* < 0.0001) than between either of these two sites with the high Cd site Xiangtan (*r* = 0.31 – 0.44, *P* < 0.001).

Among the 52 common cultivars harvested in the two years, There were significant correlations in grain Cd concentration between 2014 and 2015 (Youxian, *r* = 0.81, *P* < 0.0001; Fuyang, *r* = 0.59, *P* < 0.0001). Furthermore, the low Cd cultivars selected based on the 2014 data also exhibited low Cd accumulation again in 2015.

### Arsenic Concentration in Rice Grain

**Fig. 3:**
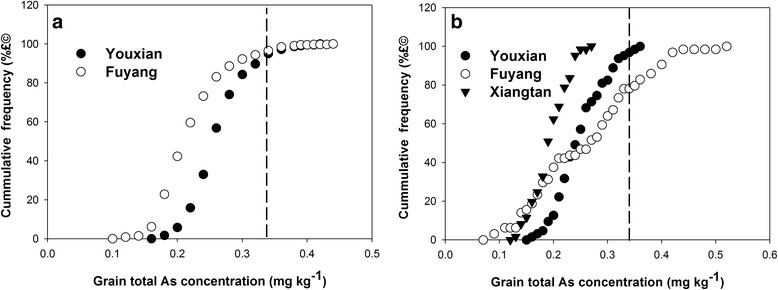
Cumulative distribution of As concentration in brown rice harvested at different sites in 2014 (**a**) and 2015 (**b**). The vertical dash line indicates Chinese Food Hygiene Standards for rice As concentration (0.2 mg kg^-1^ inorganic As, equivalent to 0.33 mg kg^-1^ total As according to the mean ratio of inorganic As to total As of 0.61)

In 2014, total As concentration in brown rice ranged from 0.11 to 0.44 mg kg^-1^ and from 0.17 to 0.42 mg kg^-1^ at Youxian and Fuyang, respectively (Fig. 3a), representing 4 and 2.5-fold variation, respectively. According to the study of Zhu et al. (2008), the mean ratio of inorganic As to total As in brown rice produced in South China is 0.61. Using this ratio, the estimated concentration of inorganic As in brown rice ranged from 0.07 to 0.27 mg kg^-1^ and from 0.10 to 0.26 mg kg^-1^ at Youxian and Fuyang, respectively. Based on this estimation, from Youxian and Fuyang site, 8 and 4% cultivars, respectively, exceeded the current Chinese Food Hygiene Standards (0.2 mg kg^-1^ inorganic As) (GB2762, 2012). The range of grain As concentration was similar between the two sites despite a 1.8 fold difference in the soil total As concentration, suggesting similar bioavailability of As in the two soils.

In 2015, total As concentration in brown rice ranged from 0.15 to 0.36 mg kg^-1^ at Youxian, from 0.07 to 0.50 mg kg^-1^ at Fuyang, and from 0.13 to 0.27 mg kg^-1^ at Xiangtan, respectively (Fig. 3b). The variations were 2 - 7 folds among the three sites. The estimated concentrations of inorganic As in all cultivars grown at Xiangtan were below the limit, whereas 5 and 22% rice cultivars grown at Youxian and Fuyang, respectively, exceeded the limit.

Two-way ANOVA showed that As concentration in brown rice was significantly affected by genotype (G), environment (E), and G × E interactions (Table 2). Environment explained 13 and 33% of the total variation in 2014 and 2015, respectively, whilst cultivars explained 38 and 31% of the total variation in the two respective years. Between 25 and 31% of the total variation could be explained by G × E interactions.

### Stability of Grain As Concentration Across Sites and Seasons

**Fig. 4:**
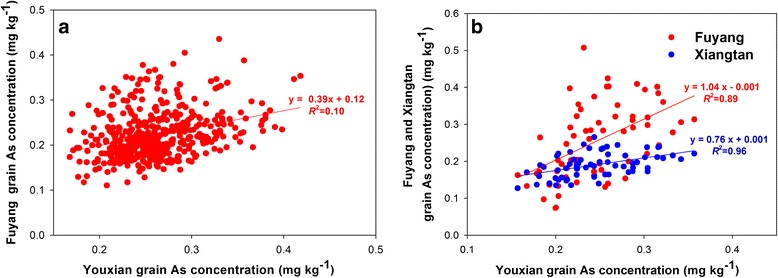
Correlation between grain As concentrations of the common cultivars grown at different sites in 2014 (**a**) and 2015 (**b**)

There were significant correlations in grain As concentration among the common cultivars between the Youxian and Fuyang sites in 2014 (*r* = 0.32, *P* < 0.0001) (Fig. 4a, Additional file 1: Table S2), and between each two of the three sites in 2015 (*r* = 0.45 – 0.51, *P* < 0.001, Fig. 4a, Additional file 1: Table S2).

Comparing the years 2014 and 2015, there were also significant correlations in grain As concentration between the two years at each site (Youxian, *r* = 0.60, *P* < 0.0001; Fuyang, *r* = 0.73, *P* < 0.0001). Five cultivars selected as low As cultivars according to the 2014 trials also exhibited low As accumulation in 2015.

### The Relationship Between Straw and Grain Cd Concentrations

**Fig. 5:**
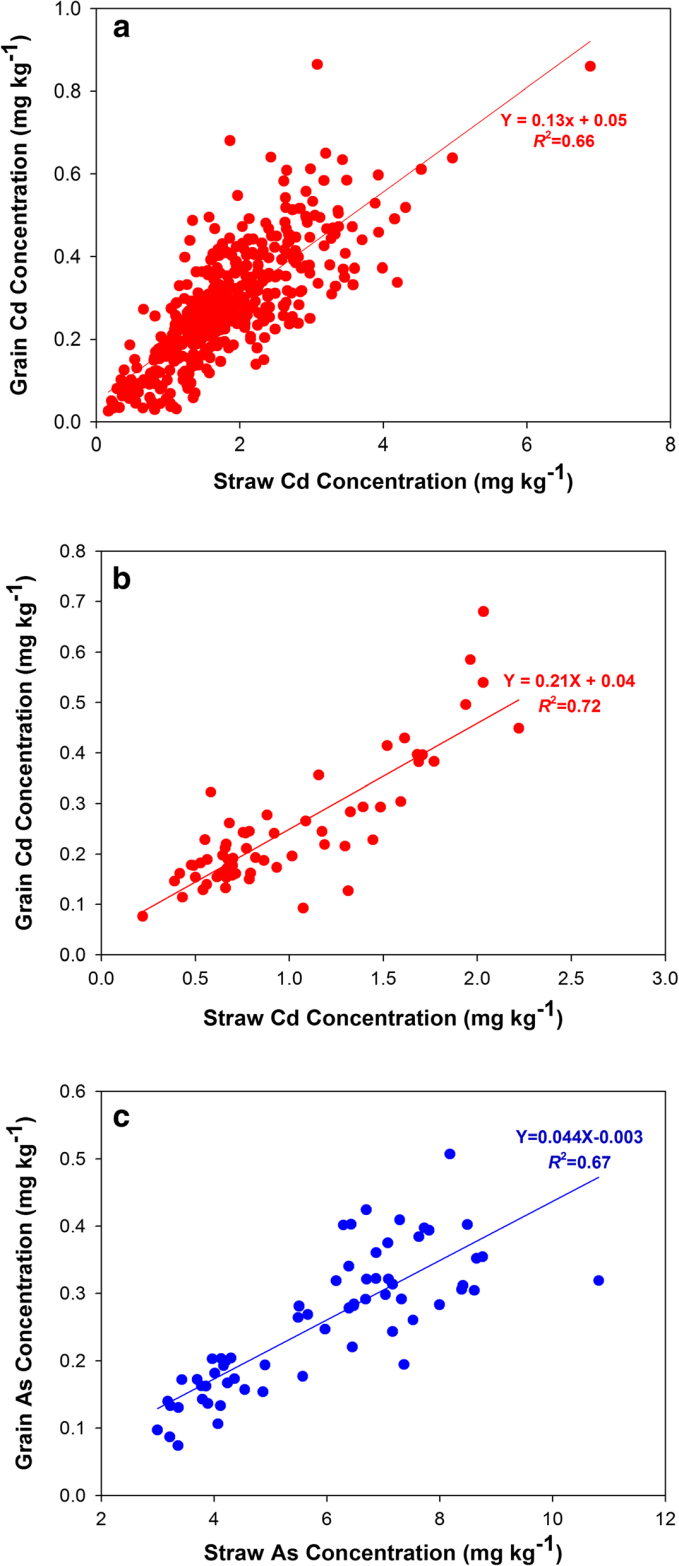
Correlation between rice straw and grain Cd concentrations at Youxian in 2014 (**a**), Fuyang in 2015 (**b**) and between rice straw and grain As concentrations at Fuyang in 2015 (**c**)

Straw samples from Youxian in 2014 and Fuyang in 2015 were collected and analyzed for Cd concentration. In both trials, there was a strong linear relationship between grain and straw Cd concentrations (Youxian, *r* = 0.78, *P* < 0.0001, n = 466, Fig. 5a; Fuyang, *r* = 0.85, *P* < 0.0001, *n* = 64; Fig. 5b). The slope of the regression was 0.13 and 0.21 in the two respective trials, which represents the mean translocation factor of Cd from the straw to the grain. Despite the strong linear relationship between grain and straw Cd concentrations, the ratio of brown Cd to straw Cd exhibited substantial genotypic variation, ranging from 0.03 to 0.42 among the 466 cultivars in the Youxian trial and from 0.008 to 0.23 among the 64 cultivars in the Fuyang trial.

Straw samples from Fuyang in 2015 were also analyzed for As concentration. There was a strong linear relationship between grain and straw As concentrations, with the slope of the regression being 0.04 (*r* = 0.82, *P* < 0.0001, *n* = 64; Fig. 5c). The slope was much lower than that for Cd, indicating a lower translocation of As from the straw to the grain than Cd. There was approximately 3 fold variation in the ratio of brown As to straw As (0.022 – 0.064) among the 64 cultivars.

### Grain Yield and the Correlations With As or Cd Concentration in Rice Grain

Grain yield of each cultivar was estimated in the Youxian trial in 2015. Among the 64 cultivars, grain yield ranged from 6018 to 10186 kg ha^-1^. There was a significant and positive correlation between grain yield and grain Cd concentration (*r* = 0.49, *P* < 0.0001, *n* = 64; Additional file 2: Figure S1), but a negative correlation between grain yield and grain As concentration (*r* = 0.33, *P* = 0.008, *n* = 64; Additional file 2: Figure S1).

### Correlations Between Grain Cd and As Concentrations and the Heading Time

**Fig. 6:**
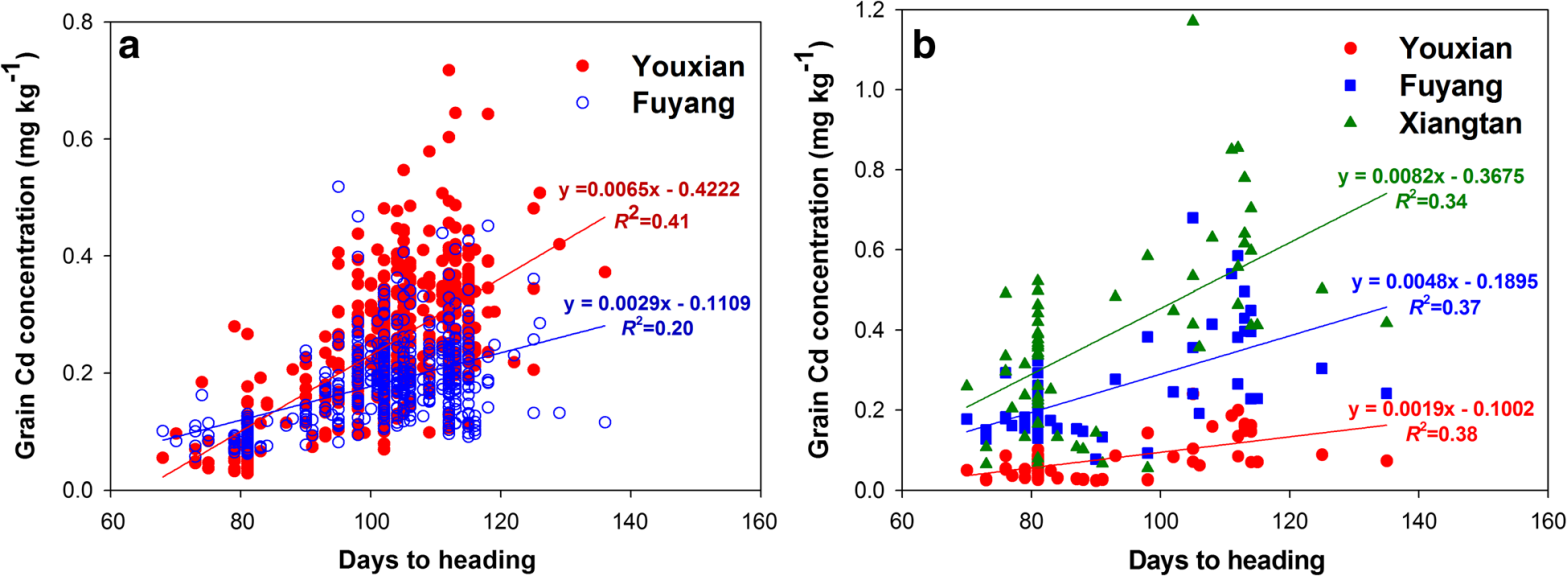
Correlation between grain Cd concentration and heading time in 2014 (**a**) and 2015 (**b**)

The days from germination to full heading varied from 68 to 136 days among the cultivars harvested in 2014. Cadmium concentration in rice grain was found to correlate significantly and positively with the heading time at both Youxian (*r* = 0.64, *P* < 0.0001) and Fuyang (*r* = 0.45, *P* < 0.0001) (Fig. 6a, Additional file 1: Table S3). Among the cultivars harvested in 2015, there were also significant correlations between the heading time (70 – 135 days) and grain Cd concentration at all three sites (*r* = 0.58 – 0.62, *P* < 0.0001, Fig. 6b, Additional file 1: Table S3).

**Fig. 7:**
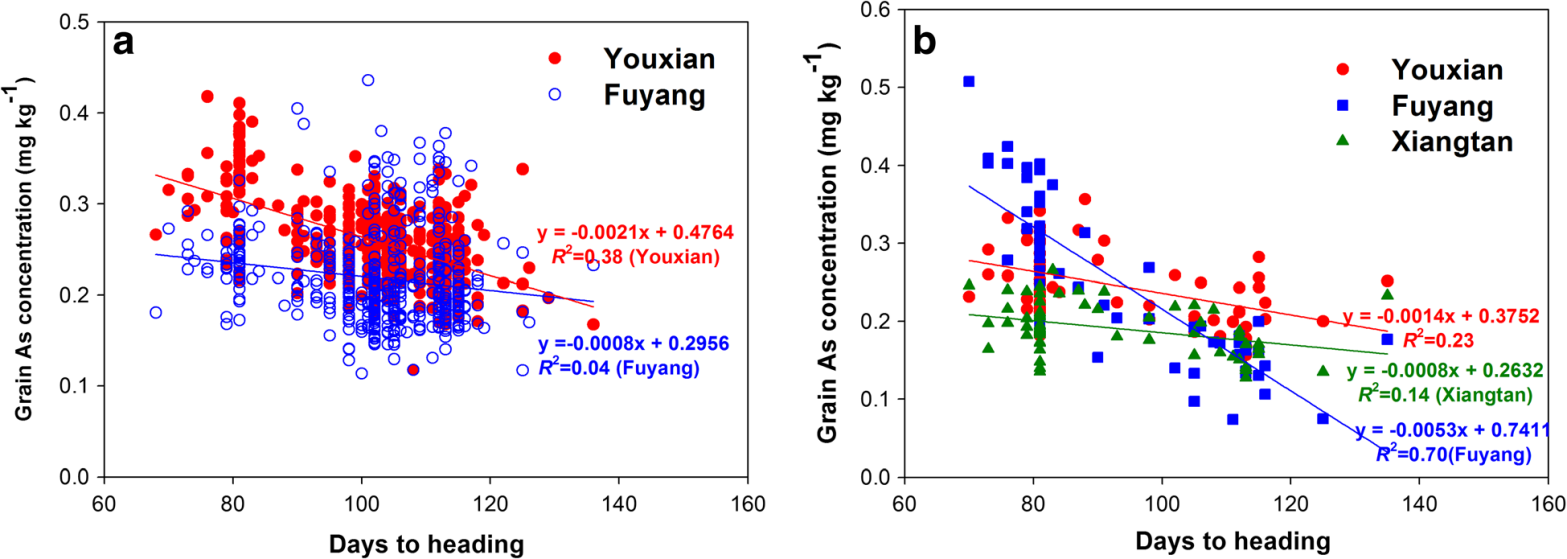
Correlation between grain As concentration and heading time in 2014 (**a**) and 2015 (**b**)

In contrast to grain Cd concentration, grain As concentration correlated significantly but negatively with the heading time. In 2014, the correlation coefficient was -0.62 (*P* < 0.0001) at Youxian site, and -0.20 (*P* < 0.0001) at Fuyang site (Fig. 7a, Additional file 1: Table S3). In 2015, there were also significant negative correlations between grain As concentrations with the heading time at all three sites (Fig. 7b, Additional file 1: Table S3).

Cadmium and As concentration in brown rice of different rice cultivars correlated significantly but negatively. In 2014, the correlation coefficient was -0.49 (*P* < 0.0001) at Youxian and -0.13 (*P* =0.0043) at Fuyang (Additional file 2: Figure S2a, Additional file 1: Table S4). In 2015, significant negative correlations were obtained between grain Cd and As concentrations at all three sites (Additional file 2: Figure S2b, Additional file 1: Table S4).

### Prospective low Cd and low As Accumulating Rice Cultivars for South China

Based on the results from the 5 trials at 3 sites across 2 years, a number of cultivars with stably low Cd or low As accumulation in grain were identified (Table 3). These cultivars accumulated Cd or As in brown rice at levels that were below the Chinese Food Safety Standards (GB 2762-2012) at all five trials, even at the Xiangtan site that had a relatively high total Cd concentration and a high Cd availability in the soil. Eight cultivars (ShenYou957, LongPing602, Weiyou402, WeiYou463, ZhuLiangYou168, T-You535, JieFengYou1 and I-You 899) and 6 cultivars (YongYou17, YongYou538, Y-LiangYou1998, II-You310, GangYou94-11 and II-You936) were identified as low Cd or low As cultivars, respectively. However, no cultivars could be considered to be low at both As and Cd. The low Cd cultivars had shorter heading time (80–90 days) than the low As cultivars (110 – 130 days).

**Table 3:** Low Cd or low As accumulating rice cultivars selected based on multiple site and season trials

Low Cd cultivars	Heading days	2014 Youxian	2015 Youxian		2014 Fuyang	2015 Fuyang	2015 Xiangtan
		Grain Cd (mg kg^-1^)	Grain Cd (mg kg^-1^)	Yield (kg ha^-1^)	Grain Cd (mg kg^-1^)	Grain Cd (mg kg^-1^)	Grain Cd (mg kg^-1^)
ShenYou957	91	0.07	0.03	9275	0.09	0.09	0.06
LongPing602	98	0.09	0.03	9515	0.15	0.13	0.10
T-You535	84	0.15	0.03	8058	0.08	0.19	0.06
JieFengYou1	87	0.12	0.03	8147	0.11	0.19	0.11
I-You899	81	0.07	0.07	7226	0.06	0.15	0.17
WeiYou402	88	n.g.	0.03	8071	n.g.	0.15	0.06
WeiUou463	83	n.g.	0.05	8923	n.g.	0.15	0.06
ZhuLiangYou168	81	0.04	0.05	7935	0.06	0.16	0.13
Low As cultivars	Heading days	Grain As (mg kg^-1^)	Grain As (mg kg^-1^)	Yield (kg ha^-1^)	Grain As (mg kg^-1^)	Grain As (mg kg^-1^)	Grain (mg kg^-1^)
YongYou17	125	0. 18	0.2	7192	0.12	0.07	0.13
YongYou538	123	0.19	0.19	8675	0.16	0.14	0.14
GangYou94-11	113	0.18	0.18	9565	0.16	0.17	0.14
Y-LiangYou1998	112	0.17	0.16	8741	0.16	0.16	0.13
II-You936	115	0.18	0.19	9549	0.15	0.10	0.18
II-You310	113	0.20	0.17	10072	0.17	0.13	0.17

n.g. not grown

## Discussion

There have been a number of field studies on the genotypic variations of As and Cd concentrations in rice grain (Norton et al. 2009; 2012a; Kuramata et al., 2013; Pinson et al., 2015). However, most of these studies tested rice germplasm resources, and those accessions found to have low levels of As or Cd accumulation may not be suitable to the growth conditions in South China, where the problem of heavy metal and metalloid contamination is serious. In the present study, we screened a large number of rice cultivars that are high yielding and widely grown in South China. The results show a large genotypic variation in grain Cd concentration, exhibiting 10 – 32 fold variation. In contrast, genotypic variation in grain As concentration was much smaller among the cultivars tested (2.5 – 4 fold). A number of cultivars with stably low accumulation of either Cd or As in the grain were identified based on the trials conducted in multiple sites and seasons (Table 3). Grain Cd or As concentrations of these cultivars grown in paddy soils contaminated with moderate levels of Cd and As were below the maximum permissible limits of China (GB2762, 2012). Even at the Xiangtan site, which had a relatively high Cd level and a low soil pH (Table 1), these selected cultivars were still below the Cd limit. Therefore, these cultivars can be immediately usable in South China to control Cd and As in rice grain.

We found that grain Cd and As concentrations correlated significantly, but in opposite ways, with the heading time. Cultivars with early heading (<90 days) tended to exhibit low Cd accumulation, whereas cultivars with late heading (>110 days) tended to exhibit low As accumulation (Fig. 5 and 6). Similarly, Sun et al. (2016) found that early flowering cultivars accumulated significantly lower Cd in the grain than later flowering cultivars. Norton et al. (2012b) reported a negative correlation between grain As concentration and flowering time in a recombinant inbred line (RIL) population derived from a cross between Bala, an Indica variety, and Azucena, a Japonica variety. Norton et al. (2012a) also found a co-localization of QTLs for flowering time and grain As concentration on chromosomes 8 and 10. When grain As data were adjusted against the heading date data, the two grain As QTLs were no longer detected, suggesting that the two traits are linked. However, whether this linkage is underpinned by physiology remains unclear. It is possible that both grain As and Cd concentrations are influenced indirectly by heading time via soil chemistry, which strongly depends on the paddy water management. It is well known that flooding of paddy soil reduces Cd bioavailability but increases As bioavailability (Xu et al. 2008; Li et al. 2009; Arao et al. 2009; Zhao et al. 2015). Draining paddy water during the later phase of grain filling is a normal agronomic practice for rice cultivation. Therefore, cultivars with early heading time might have experienced flooded conditions during most of the grain filling period, resulting in low Cd and high As bioavailability in the soil, and hence low Cd and high As accumulation in the grain. In contrast, cultivars with late heading might have encountered drained soil conditions during the grain filling period, leading to high Cd and low As bioavailability in the soil, and hence high Cd and low As accumulation in the grain. The opposite effect of water management on As and Cd availability in soil may also explain the negative correlation between As and Cd concentrations in rice grain (Additional file 2: Figure S2, Additional file 1: Table S4). In 2015, cultivars were separated into early, middle and late heading groups and planted into 3 subplots within each replicate at two sites (Youxian and Xiangtan) to allow paddy water management to be tailored according to heading time. The positive and negative relationships between heading date and grain Cd and As, respectively, were still apparent in these two trials (Figs. 6 and 7), suggesting possible physiological mechanisms underlying these relationships. The negative correlation between grain Cd and As concentrations suggests that it would be difficult to find cultivars with low accumulation of both Cd and As. One way to circumvent this dilemma has recently been tested by Ishikawa et al. (2016), who grew a rice mutant with nonfunctional OsNramp5 under aerobic soil conditions and achieved low accumulation of both Cd and As in grain.

In addition to environmental factors, rice genotype is a key factor controlling As and Cd accumulation in rice grain (Norton et al., 2009; Pinson et al., 2015). A number of QTLs have been reported to be associated with rice grain As accumulation (Zhang et al., 2008; Norton et al., 2012a; Kuramata et al., 2013; Pinson et al., 2015) or Cd accumulation (Ishikawa et al. 2005; Ueno et al., 2009; Zhang et al., 2011; Abe et al., 2013). However, so far only a QTL for shoot and grain Cd concentration located on the chromosome 7 has been cloned, with *OsHMA3* as the causal gene explaining the genotypic variation (Ueno et al. 2010; Miyadate et al. 2011). This gene encodes a Cd transporter localized on the tonoplast functioning to sequester Cd in the vacuoles of root cells, thus limiting Cd translocation to the shoots and grain (Ueno et al. 2010; Miyadate et al. 2011). Several loss-of-function alleles of OsHMA3 have been identified in a number of Indica (Ueno et al. 2010; Miyadate et al. 2011) and Japonica (Yan et al., 2016) cultivars, leading to high accumulation of Cd in rice grain. However, these loss-of-function alleles are rare among collections of rice cultivars and germplasm (Yan et al. 2016). It is possible that other functional alleles of OsHMA3 may vary in the functionality by exhibiting different transport activities for Cd. This possibility remains to be tested. The strong linear relationship between straw and brown rice Cd or As concentrations (Fig. 5) is consistent with the suggestion that the root to shoot translocation of Cd and As is the key process controlling the accumulation of Cd or As in rice grain (Uraguchi et al. 2009; Zhao et al., 2009). However, there was also substantial variation in the ratio of brown rice Cd or As to straw Cd or As concentrations, suggesting genotypic variation in the translocation from leaves and stems to rice grain. In addition, the ratio of brown rice Cd to straw Cd concentration was much higher than that of As, indicating that Cd is more mobile during translocation from straw to grain.

A number of other genes are known to be involved in Cd or As uptake and translocation in rice, but it remains unknown if there are allelic variations of these genes that can explain the variations in grain Cd or As accumulation. Examples include OsNramp5 responsible for the uptake of Cd into the root cells (Ishikawa et al., 2012; Sasaki et al. 2012; Yang et al. 2014), OsHMA2 and OsLCT1 for Cd re-distribution between rice tissues (Uraguchi et al. 2011; Satoh-Nagasawa et al. 2012), OsLsi1 and OsLsi2 for arsenite uptake into rice roots (Ma et al. 2008), OsABCC1 for arsenite sequestration into the vacuoles (Song et al., 2010), OsHAC1;1 and OsHAC1;2 for arsenate reduction to allow subsequent arsenite efflux (Shi et al. 2016), and plant inositol transporters for arsenite loading to the phloem and regulating As accumulation in the seeds of *Arabidopsis thaliana* (Duan et al., 2016). Identification of alleles that can limit Cd or As accumulation in the grain is prerequisite for breeding low accumulation cultivars using marker assisted selection approach.

It has been reported that Indica rice cultivars tend to accumulate higher levels of Cd in shoots and grain than Japonica cultivars (Liu et al. 2005; He et al. 2006; Uraguchi & Fujiwara 2013; Sun et al. 2016). However, no significant difference between Indica and Japonica cultivars for either As or Cd concentration in the grain was found in the present study (Additional file 2: Figure S3). There are large genotypic variations among both Japonica and Indica cultivars (Ueno et al., 2009). Pinson et al. (2015) and Yan et al. (2016) showed that some Japonica cultivars can accumulate high Cd concentrations. Therefore, depending on the cultivars tested, Japonica cultivars as a group may not always show a significant lower Cd accumulation than the Indica group. It has also been claimed that hybrid rice can accumulate more Cd and As than non-hybrids (Gong et al., 2006; Rahman et al. 2007). However, in this study, there was no significant difference between hybrid and non-hybrid cultivars in As or Cd concentrations in brown rice (Additional file 2: Figure S3). A recent study by Sun et al. (2016) also showed no significant difference in grain Cd concentration between Indica hybrids and Indica inbred cultivars. It is the genetic diversity rather than the type of hybrid versus inbred that determines the relative Cd accumulation among rice cultivars (Sun et al. 2016).

## Conclusions

For both grain Cd and As concentrations, there were significant G x E interactions in the present study. Despite these interactions, the results show that it is possible to identify locally adapted rice cultivars with low accumulation of Cd or As in the grain with the trait being stable across multiple sites and seasons. These cultivars (or their hybrid parents) can be valuable materials for investigating the genetics underpinning low accumulation of As and Cd. However, it is difficult to select cultivars with low accumulation of both Cd and As.

## Methods

### Rice Cultivars

In 2014, 471 rice cultivars (Additional file 1: Table S1) were grown at two field sites contaminated with moderate levels of Cd. These cultivars are commonly grown in large acreage in southern China, with approximately 300 cultivars being the main cultivars in Hunan province, where Cd and As contamination in paddy soils are widespread. The majority (425) of the cultivars are Indica rice, among which 408 and 17 are hybrid and conventional cultivars, respectively. The remainders (46) are conventional Japonica cultivars. The days to heading of these cultivars varied from 68 to 136 days (from germination to full heading).

Base on the results of the trials in 2014, 52 rice cultivars were selected for further trials at three sites in 2015. These included 44 cultivars with low grain Cd concentrations, 3 cultivars with high Cd concentrations, and 5 cultivars with low As concentrations. Because most of the 52 selected cultivars are relatively early heading, 11 additional main rice cultivars with late heading were also included in the trials in 2015, giving rise to a total number of 63 rice cultivars (Additional file 1: Table S1).

### Field Sites

The field experimental sites in 2014 were located in Youxian county, Hunan province (latitude 27°08‘; longitude 113°22’) and in the Fuyang district of Hangzhou city, Zhejiang province (latitude 30°07’; longitude 119°95’). The total Cd concentrations in the paddy soils were around 0.5 and 0.4 mg kg^-1^ for Youxian and Fuyang sites, respectively (Table 1), both exceeding the Cd limit of the Chinese soil environmental quality standard (0.3 mg kg^-1^ for soils with pH < 7.5) (GB 15618, 1995). The total soil As concentrations were about 20 and 12 mg kg^-1^ for Youxian and Fuyang sites, respectively (Table 1), both were higher than the background level of As (<10 mg kg^-1^), but still below the As limit of the Chinese soil environmental quality standard (30 mg kg^-1^ for paddy soils) (GB 15618, 1995). The soils at the Youxian Fuyang site are acidic (pH 4.9 - 5.6). In 2015, field experiments were conducted at three sites, including the two sites used in 2014, and an additional site at Xiangtan city, Hunan province (latitude 27°83’; longitude 112°91’). At Xiangtan site, the soil is also acidic (pH 4.9) and contains a higher level of Cd (1.4 mg kg^-1^) than the other sites, but a similar level of total As (19 mg kg^-1^).

### Rice Cultivation

Single-cropping rice cultivation was conducted in both 2014 and 2015. Seeds were germinated and sown on seedbeds in early May, and transplanted to the field plots in early June. Seedlings of different cultivars were transplanted in randomized block design with 3 replicates. In each replicate, each genotype was planted in 3 (in 2014) or 20 rows (in 2015) of 10 hills (1 seedling per hill). The distance between row and hills was 20 cm. To separate different cultivars, 40 cm unplanted area was included between adjacent cultivars. Paddy field water was managed according to the local practices, with paddy fields being flooded during the rice growth season except the late tillering stage and one week before harvesting when water was drained. In 2015 at Youxian and Xiangtan sites, cultivars were grouped into early, middle and late heading groups, which were planted into 3 subplots within each replicate to allow water management of each subplot depending on the heading time. Compound fertilizers (16% N, 16% P_2_O_5_, 16% K_2_O) were applied at three times, 200, 100 and 100 kg/ha, respectively, at one day before transplanting and 10 and 30 days after transplanting. In the two latter applications, 60 kg/ha of urea was also applied. Fungicides and pesticides were applied according to the local practices for rice crops. Plants of each cultivar were harvested 30 days after full heading, between August and October. Grain and straw from the central 6 hills were harvested and pooled together for elemental analysis. Plants were cut from about 20 cm above the soil surface and placed in a nylon mesh bag. Samples were dried under the sunlight and then separated into grain and straw.

Grain yield of each cultivar was estimated at Youxian in 2015. Approximately 100 hills were harvested from each replicate plot of each cultivar. Grain were dried and weighed. Grain yield was calculated by using the planting density of 250000 hills per hectare.

### Plant and Soil Analysis

Soil samples were taken before transplanting. From each field, three composite soil samples were collected from the three blocks of the experiment, each consisting of five cores randomly taken from the 0–20 cm depth within the block. Soil samples were air-dried and crushed to pass through a 2-mm nylon sieve. A portion of each soil sample was ground with an agate grinder to pass through a 0.15 mm nylon sieve. For the determination of the concentrations of Cd, As and other heavy metals, 0.2 g finely ground soil sample was weighed into a quartz glass tube, to which 2.5 ml of high purity nitric acid was added and left to stand overnight. Hydrogen peroxide (2.5 ml, 30%) was then added and the sample was digested on a block digester at 100 °C for 1 h, 120 °C for 1 h, and 140 °C for 4 h. The concentrations of Cd and As in the digest solution were determined by inductively coupled plasma mass spectrometry (ICP-MS; Perkin-Elmer Nexion 300x). For quality control, a certified reference soil (GBW 07405, China Standard Materials Research Center, Beijing, China) was included in the analysis. The average recovery of for Cd and As ranged from 91 to 105% and from 97 to 103%, respectively. Soil pH was determined using a combined glass electrode in a suspension of soil (<2 mm) and deionized water (1:2.5, w/v).

Grain samples were separated into husks and brown (unpolished) rice using a de-husking machine (JLGJ45, Taizhou Food Instrument Factory, Taizhou, China). Brown rice and straw samples were oven-dried at 65 °C for 3 days, and ground to fine powders using a mill (JNMJ3, Taizhou Food Instrument Factory, Taizhou, China). Plant samples (0.2 g of rice grain and 0.1 g of straw) were digested with 2 ml of high purity nitric acid in a microwave digestion oven (Mars 5, CEM Corporation, USA) using the following program, 55 °C for 10 min, 75 °C for 10 min, and 95 °C for 30 min. After cooling, the remaining acid was evaporated and the digests were dissolved in 30 mL 2% HNO_3_. The concentrations of Cd and As in the digest solutions were determined by ICP-MS (Perkin-Elmer Nexion 300x). For quality control, a certified reference material (rice flour GBW-10010, China Standard Materials Research Center, Beijing, China) was included in the analysis. The average recovery for Cd and As ranged from 89 to 102% and from 92 to 105%, respectively.

### Statistical Analysis

All data were subjected to two-way analysis of variance (ANOVA) followed by Student-Newman-Keuls multiple comparisons using windows-based SPSS 11.5. Data presented are means ± SD (*n* = 3). Curve fitting was performed using SigmaPlot 10.0.

## Additional Files

Additional file 1: List of rice cultivars used for this study. (XLSX 22 kb)

Additional file 2: Boxplots of grain Cd and As concentrations of different rice subgroups, and correlation of grain As and Cd concentrations and with grain yield respectively. (DOCX 251 kb)
