# Supplementary material for: Genotypic and Environmental Variations in Grain Cadmium and Arsenic Concentrations Among a Panel of High Yielding Rice Cultivars
Source: Rice (N Y). 2017 Mar 28;10:9. doi: 10.1186/s12284-017-0149-2 (PMC5371165; doi:10.1186/s12284-017-0149-2)
Supplement: Supplementary file 2 — Boxplots of grain Cd and As concentrations of different rice subgroups, and correlation of grain As and Cd concentrations and with grain yield respectively. (DOCX 251 kb) [file 12284_2017_149_MOESM2_ESM.docx]

**Supplementary Information**

Figure S1. Correlation between grain yield and grain As or Cd concentration at Youxian in 2015.

Figure S2. Correlation between grain As and Cd concentrations in 2014 (a) and 2015 (b).

Figure S3. Boxplots of grain Cd (a) and As (b) concentrations of rice cultivars grown in 2014, grouped into hybrid, non-hybrid Indica and non-hybrid Japonica. Statistical analysis showed no significant difference between groups for either grain Cd or grain As concentrations.

Table S2. Linear regression of Cd and As concentration in rice grains harvested between different sites.

| Year | Cd or | Grain concentration | | Equation | *R*^2^ | *P* | n |
| --- | --- | --- | --- | --- | --- | --- | --- |
|  | As | y | x |  |  |  |  |
| 2014 | Cd | Fuyang | Youxian | y = 0.28x + 0.10 | 0.30 | <0.0001 | 456 |
|  | As | Fuyang | Youxian | y = 0.39x + 0.12 | 0.10 | <0.0001 | 456 |
| 2015 |  | Fuyang | Youxian | y = 1.88 x + 0.10 | 0.56 | <0.0001 | 60 |
|  | Cd | Xiangtan | Youxian | y = 1.30 x + 0.28 | 0.10 | <0.0001 |  |
|  |  | Xiangtan | Fuyang | y = 0.79 x + 0.19 | 0.19 | <0.0001 |  |
|  |  | Fuyang | Youxian | y = 1.04 x - 0.001 | 0.89 | <0.0001 | 60 |
|  | As | Xiangtan | Youxian | y = 0.76 x + 0.001 | 0.96 | <0.0001 |  |
|  |  | Xiangtan | Fuyang | y = 0.66 x + 0.003 | 0.88 | <0.0001 |  |

Table S3. Linear regression of Cd and As concentration in rice grains with rice heading time (days).

| Year | Site | Cd or As | Equation  y (grain Cd/As concentration)  x (days to heading) | *R*^2^ | *P* | n |
| --- | --- | --- | --- | --- | --- | --- |
| 2014 | Youxian | Cd | y =0.0065x - 0.4222 | 0.41 | <0.0001 | 466 |
|  |  | As | y = -0.0021x + 0.4764 | 0.38 | <0.0001 |  |
|  | Fuyang | Cd | y = 0.0029x - 0.1109 | 0.20 | <0.0001 | 462 |
|  |  | As | y =-0.0008x + 0.2956 | 0.04 | <0.0001 |  |
| 2015 | Youxian | Cd | y = 0.0019x - 0.1002 | 0.38 | <0.0001 | 62 |
|  |  | As | y = -0.0014x + 0.3752 | 0.23 | <0.0001 |  |
|  | Fuyang | Cd | y = 0.0048x - 0.1895 | 0.37 | <0.0001 | 63 |
|  |  | As | y = -0.0053x + 0.7411 | 0.70 | <0.0001 |  |
|  | Xiangtan | Cd | y = 0.0082x - 0.3675 | 0.34 | <0.0001 | 61 |
|  |  | As | y = -0.0008x + 0.2632 | 0.14 | 0.0028 |  |

Table S4. Linear regression between Cd and As concentrations in rice grains.

| Year | Site | Equation  y (As concentration)  x (Cd concentration) | *R*^2^ | *P* | n |
| --- | --- | --- | --- | --- | --- |
| 2014 | Youxian | y = -0.1857x + 0.3046 | 0.24 | <0.0001 | 466 |
|  | Fuyang | y = -0.0992x + 0.2364 | 0.02 | 0.0043 | 462 |
| 2015 | Youxian | y = -0.4857x + 0.2863 | 0.27 | <0.0001 | 62 |
|  | Fuyang | y = -0.6836x + 0.4272 | 0.30 | <0.0001 | 63 |
|  | Xiangtan | y = -0.0534x + 0.2127 | 0.13 | 0.0046 | 61 |
